# How Bacterial Redox Sensors Transmit Redox Signals via Structural Changes

**DOI:** 10.3390/antiox10040502

**Published:** 2021-03-24

**Authors:** In-Gyun Lee, Bong-Jin Lee

**Affiliations:** 1 Chemical Kinomics Research Center, Korea Institute of Science and Technology (KIST), 5 Hwarangro 14-gil, Seongbuk-gu, Seoul 02792, Korea; ik86@kist.re.kr; 2 Research Institute of Pharmaceutical Sciences, College of Pharmacy, Seoul National University, Seoul 08826, Korea

**Keywords:** bacterial redox sensors, oxidative stress, structural biology

## Abstract

Bacteria, like humans, face diverse kinds of stress during life. Oxidative stress, which is produced by cellular metabolism and environmental factors, can significantly damage cellular macromolecules, ultimately negatively affecting the normal growth of the cell. Therefore, bacteria have evolved a number of protective strategies to defend themselves and respond to imposed stress by changing the expression pattern of genes whose products are required to convert harmful oxidants into harmless products. Structural biology combined with biochemical studies has revealed the mechanisms by which various bacterial redox sensor proteins recognize the cellular redox state and transform chemical information into structural signals to regulate downstream signaling pathways.

## 1. Introduction

Under physiological conditions, the cytoplasm of most bacterial cells is maintained under mildly reducing conditions to favor the proper folding and function of proteins [1,2]. Bacteria encounter diverse oxidative stress-generating species, such as reactive oxygen species (ROS), reactive nitrogen species (RNS), and reactive chlorine species (RCS), which are continuously produced inside cells (endogenous sources; as byproducts of oxidative phosphorylation, lipid metabolism, autoxidation of redox enzymes, and/or respiration) or produced by the host (exogenous sources) during the course of infections [3,4]. These oxidative stresses can have a devastating effect on the proper function of many macromolecules, including proteins, DNA, and membrane lipids, inside the bacterial cell by over-oxidizing these macromolecules [5]. Therefore, most bacteria have evolved a number of protection strategies to defend themselves with scavenging enzymes that react with harmful oxidants and convert them into harmless products [3,5,6,7,8]. To regulate the signal transduction and gene expression of these oxidative stress-induced scavenging enzymes, bacteria produce a variety of redox-sensing proteins, including transcriptional regulators, that can react with oxidative stress-generating molecules in defined ways [9]. By reacting with ROS, RNS, or RCS in unique ways, each redox sensor protein family senses various oxidative stresses and converts the chemical information into a structural change, generating a signal that can be recognized by downstream signaling effectors. For instance, redox sensors perceive redox status by exploiting the amino acids (cysteine (Cys), methionine (Met), or histidine (His)) or bound cofactors ([Fe–S] cluster, heme, flavins, and pyridine nucleotides) of redox-participating compounds. In this review, we discuss several example mechanisms by which bacterial redox sensory proteins respond to diverse oxidative stresses. We focus on unique structural changes that each redox sensor protein family undergoes upon recognizing redox stress, and discuss how these structural changes are converted into cellular responses.

## 2. Cysteine-Based Redox Sensors

ROS/RNS primarily react with cysteine residues whose oxidative modification leads to functional and structural modifications in ROS/RNS-sensing regulatory proteins. Cysteine is uniquely suited for sensing diverse redox signals, mainly because its thiol side chain can be oxidized into various reversible/irreversible states, such as a s-nitrosothiol, sulfenic acid, sulfinic acid, sulfonic acid, sulfenamide, persulfide, and various disulfides [10]. By reacting with ROS/RNS, cysteine residues function as detectors of redox status, and the subsequent chemical change of the oxidized cysteine can be translated into a protein conformational change leading to an effector response.

### 2.1. Sensing by Intramolecular Disulfide Bond Formation: OxyR

**Figure 1:**
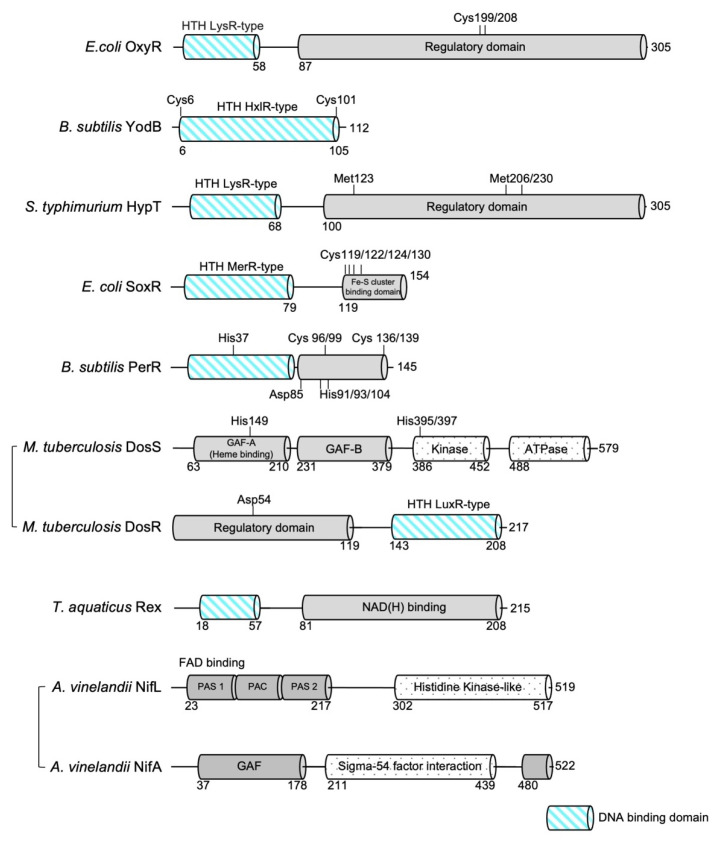
**Domain organization of bacterial redox sensors highlighting the DNA-binding domain**. Residues critical for redox sensing are marked. The diagrams of *M. tuberculosis* DosS and *A. vinelandii* NifL are not to scale with the other domain diagrams.

The OxyR transcriptional regulator senses intracellular oxidative stress in the majority of Gram-negative bacteria [11,12]. OxyR has been extensively studied in *Escherichia coli* and shown to function primarily as a global transcriptional activator of oxidative responses caused by H_2_O_2_, regulating the expression of a range of antioxidant defense genes [11,13,14]. OxyR is a member of the LysR-type transcriptional regulator (LTTR) family, composed of an N-terminal DNA-binding domain (DBD) and a C-terminal regulatory domain (RD) (Figure 1) [15]. The DBD adopts a classical winged helix-turn-helix fold consisting of three α-helices and two characteristic loops (‘wings’). The C-terminal regulatory domain, which lies in the two conserved oxidative stress-sensing cysteine residues (Cys199 and Cys208), consists of two α/β domains (RD-I and RD-II) that are linked by two interdomain strands. Similar to other LTTR family members, OxyR adopts tetrameric assembly, with dimers of RD interfaced in homodimer arrangements and the interactions between two homodimers predominated by the interactions between DBDs [16,17].

**Figure 2:**
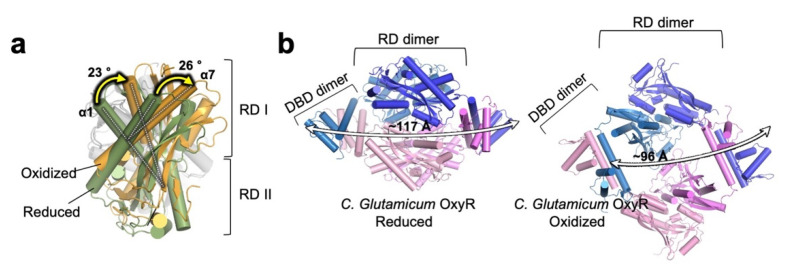
**Structural rearrangement of *C. glutamicum* OxyR upon disulfide bond formation.** (**a**) Two regulatory domain (RD) protomers within the dimer in the oxidized form (orange; PDB ID: 6G1B) have a relative rotation of ∼30°, which is not evident in the reduced state (Green; PDB ID: 6G1D). The rotation of α1 and α7 of the RD caused by disulfide bond formation is indicated. The subunit that is used as a reference for the superimposition on the backside of the dimer is colored in gray for clarity. (**b**) Reorganization of the tetrameric assembly upon disulfide bond formation. The twist motion in the dimeric interface induces structural rearrangement in the tetrameric assembly, resulting in the shortening of the distances between the DBDs of an RD homodimer (~117 Å in the reduced state (left) and ~96 Å in the oxidized state (right)). Protein Data Bank, PDB.

Crystal structures of RD and full-length OxyR in various redox states have been obtained from *E. coli*, as well as other various organisms, revealing how OxyR transfers oxidative signals through structural rearrangements (Table 1). The H_2_O_2_-dependent activation of OxyR begins with the S-hydroxylation of the H_2_O_2_ concentration-sensing conserved Cys199 in the presence of H_2_O_2_, resulting in a Cys199-SOH intermediate [17,18,19]. Although the structure of Cys199 in the S-hydroxylated form has not been elucidated yet, various biochemical studies, including circular dichroism, mass spectrometry, and fluorescence spectroscopic studies, as well as crystallographic studies of a Cys199-SOH mimicking mutant (Cys199Asp), support the idea that the hydroxylation of Cys199 forces its movement out of its small hydrophobic pocket (peroxidatic pocket) [16,19,20]. This structural change is thought to allow the ~17 Å gap separating the two reactive cysteines under reducing conditions to come into close proximity, promoting the formation of a disulfide bond. The formation of a disulfide bond induces the generation of new interactions as well as new secondary structural elements, subsequently inducing a structural change in the relative positions of the protomers of the RD homodimers. In the oxidized state, two RD protomers oriented antiparallel within the dimer have a relative rotation of ~30° that is not evident in the reduced state [16] (Figure 2a). This twist motion within the RD dimers induced by the formation of a disulfide bond affects the tetrameric assembly of the protein. The twist motion induces an asymmetrical hinge motion of the flexible loop connecting DBDs with RDs, while the interactions between DBDs mediating the interprotomer interactions remain essentially the same (Figure 2b). As a result, the distances between the DBDs of an RD homodimer are significantly shortened, yielding an altered DNA-binding topology and affinity [17,19]. Although the structural modeling and mutational analysis studies revealed the important residues involved in protein-DNA interactions [21,22], further structural analysis of full-length DNA-bound OxyR may reveal the atomic details.

### 2.2. Sensing by Irreversible Thiol S-Alkylation: YodB

**Figure 3:**
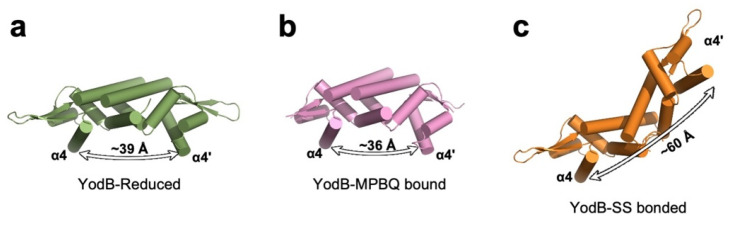
**Distinct structural rearrangement of *B. subtilis* YodB upon S-alkylation and disulfide bond formation.** The overall structure of YodB in the reduced state (panel (**a**); PDB ID: 5HS7), methyl-*p*-benzoquinone (MPBQ)-bound (panel (**b**); PDB ID: 5HS9) and with disulfide-bonds (panel (**c**); PDB ID: 5HS8), is shown. The distances between DNA major groove recognition helices (α4) in each dimer are shown.

*Bacillus subtilis* YodB belongs to the MarR/DUF24 family of transcriptional regulators that sense and respond to various reactive electrophilic species, including diamide, quinones, and aldehydes [23,24]. In contrast to most other ROS-generating molecules, quinone molecules can act as electrophiles and form irreversible S-adducts with cellular thiols through reactions involving a reductive Michael-type addition, and induce the production of ROS such as superoxide anions [25,26]. The Gram-positive bacterium *B. subtilis* responds to toxic quinone compounds by using the specific stress response regulator YodB [23]. Similar to most other prokaryotic transcription factors that utilize an HTH motif to bind DNA, YodB functions as a homodimer, and each subunit consists of 112 amino acids containing two reactive cysteine residues critical for sensing oxidative stresses, as revealed by transcriptome and proteome analysis [23,27] (Figure 1). YodB is uniquely suited for sensing diverse ROS-generating molecules ranging from diamide to quinone molecules, as its reactive cysteine residues (Cys6 and Cys101) can form irreversible S-adducts and disulfide bonds, causing distinct structural changes [28]. Of the two oxidative stress-responsive cysteine residues, Cys6 is more reactive towards electrophiles, as Cys6 is located at the N-terminus of an a-helix, and a helix dipole moment activates the nucleophilicity of the sulfhydryl group (-SH) of cysteine [29]. More reactive Cys6 can form an irreversible S-alkylation adduct with quinones. X-ray crystallographic and NMR studies revealed that the covalent binding of a quinone species (methyl-*p*-benzoquinone, MPBQ) to Cys6 does not substantially alter the conformation of the overall YodB dimer compared with the change acquired in the reduced state (overall root mean square deviation ( r.m.s.d.) of 1.6 Å) [28] (Table 1, Figure 3a,b). However, MPBQ binding produces a shift in DNA-binding helices (~3 A), which move closer to each other within the YodB dimer. The structural change is likely sufficient to alter the DNA-binding affinity of YodB toward its cognate DNA, as YodB–DNA binding would cause a strong steric clash with the DNA major groove.

In contrast to quinone molecules that form irreversible S-adducts with YodB Cys6, ROS-generating diamide induces the formation of reversible intermolecular disulfide bonds (Cys6-Cys101′ and Cys6′-Cys101, where prime denotes the other subunit). The formation of disulfide bonds produces overall structural changes in the YodB dimer that make it structurally distinct from the quinone-bound YodB dimer [28] (Figure 3c). One of the most striking features observed in the disulfide-bonded YodB dimer is the substantial movement of the DNA-recognition helices (α4 and α4′). The distance between α4 and α4′ (between Lys48/Lys48′-Cα atoms) in YodB_reduced_ is ∼39 Å, whereas that between equivalent Lys48-Cα pairs in YodB_diamide_ is ∼60 Å. Although the overall structural rearrangements observed in YodB_diamide_ were more substantial, the in vitro fluorescence polarization assay showed that the resulting affinity change towards its cognate DNA was minimal compared to that of YodB_MBPQ_. YodB binds to the promoter region of *azoR1* with a preference for a palindromic sequence separated by ~7 bp (TACT [7] AGTA), suggesting that each HTH motif within the dimer interacts with two consecutive major grooves, which is similar to the QsrR-DNA structure [23,30]. Upon the formation of disulfide bonds, YodB is likely unable to interact with one major groove, as reflected by the reduction in overall affinity, but can remain bound to the other major groove, as the distance between the recognized DNA recognition helices is too great to cause steric hindrance with each other. In contrast, MPBQ binding shortens the distance between the recognition helices, by only ~3 Å, and the two HTH motifs no longer fit the two consecutive major grooves, causing YodB to dissociate from DNA [28]. Overall, *B. subtilis* has evolved to discriminate various redox signals using a single transcriptional regulator, YodB, to respond to various toxic electrophile- and ROS-generating compounds more efficiently than would be realized by expressing each specific transcriptional regulator that senses various redox signals.

## 3. Methionine-Based Redox Sensors

### Sensing by Methionine Oxidation: HypT

Although cysteine residues frequently serve as redox sensors, sensing is not always mediated by cysteine; oxidative stresses can also be sensed by methionine residues. Neutrophils in humans and other higher eukaryotes produce the reactive oxygen species hypochlorite (HOCl) to kill invading microorganisms [31]. The hypochlorite-specific transcription factor HypT belongs to the LTTR family and has been shown to protect *E. coli* from HOCl stress [32,33]. HypT specifically recognizes HOCl and is not activated upon H_2_O_2_- or diamide-induced stress, and regulates the expression of diverse genes involved in HOCl detoxification through conserved methionine residues that serve as sensors for HOCl-induced stress [34,35]. The oxidation of three conserved methionine residues (Met123, Met206, and Met230) to methionine sulfoxides has been shown to increase the viability of *E. coli* under HOCl-mediated oxidative stress [34,35]. Since its first functional identification in *E. coli*, HypT has also been identified in *Salmonella typhimurium,* and the high-resolution structure of *S. typhimurium* HypT revealed the molecular details by which HypT methionine residues sense HOCl molecules, and how external oxidative stresses are translated into structural rearrangements to regulate the expressions of downstream target genes [33].

**Figure 4:**
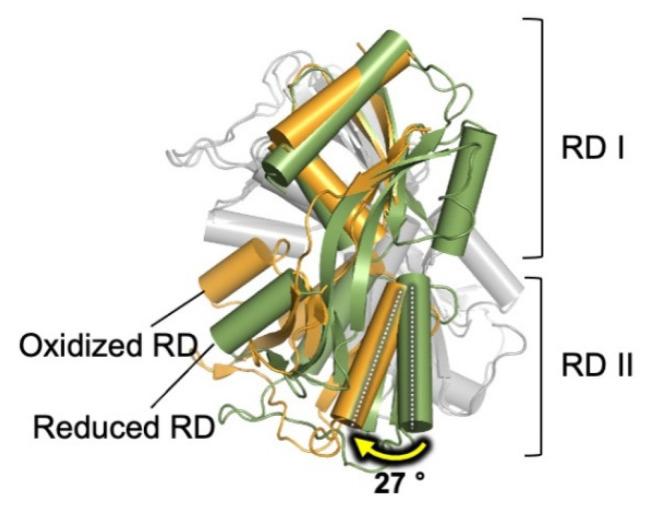
**Reorganization of the dimeric assembly of the *S. typhimurium* RD upon methionine oxidation.** Two RD protomers within the dimer in the oxidized form (orange; PDB ID: 5YEZ) have a relative rotation of ∼30° in RD II compared with that in the reduced state (green; PDB ID: 5YDO). The rotation of α8 in RD II caused by oxidation is indicated. The subunit, which is used as a reference for the superimposition, on the backside of the dimer is colored in gray for clarity.

Similar to other LTTR-type transcription factors, *S. typhimurium* HypT is also composed of an N-terminal DBD and a C-terminal regulatory domain (RD) connected by a flexible linker domain (Figure 1). The structures of each DBD and RD exhibit high structural similarity to LTTR family proteins. The overall structure and dimeric assembly of the RDs in *S. typhimurium* HypT are very similar to those of *E. coli* OxyR, one of the representative members of the LTTR family (r.m.s.d. of 4.3 Å for 192 equivalent Ca atoms between the RD of *E. coli* OxyR (PDB ID: 1I69; chain A) and the RD of *S. typhimurium* HypT chain A (PDB ID: 5YDO; chain A)) [16,17,19,36,37,38] (Table 1, Figure 4). The overall structure, and hence the dimeric assembly, of the DBD domains in *S. typhimurium* show similarity to those of *E. coli* and *Pseudomonas aeruginosa* OxyR (r.m.s.d. of 1.7 Å for 80 equivalent Cα atoms between the DBD of *P. aeruginosa* OxyR (PDB ID: 1I69; chain A) and the RD of *S. typhimurium* HypT (PDB ID: 5YDO]; chain A). However, noticeable differences are observed in domain arrangements when the structures of the full-length OxyR and HypT proteins are compared, although they both adopt a dimer-of-dimers tetrameric assembly [19,33]. In the structure of full-length *P. aeruginosa* OxyR, the two DBD domains are highly asymmetric, as one DBD adopts an extended conformation whereas the other DBD is tightly packed against its own RD subunit. Compared to this arrangements, the DBDs of *S. typhimurium* HypT are arranged in a highly symmetrical way, with all four DBDs facing inward upon interaction with the other three RD subunits, implying that the mechanism of transcriptional regulation of HypT by oxidative stress may differ from that of other LTTR-type transcription factors [19,33].

For the full activation of HypT, three conserved Met residues (Met123, Met206, and Met230) must be oxidized, and Met206 seems to be the most critical of the three residues [39]. Interestingly, *S. typhimurium* Met206 resides within the ligand-accessible small hydrophobic cavity between subdomains RD I and RD II, analogous to the H2O2 molecule-sensing Cys199 in *E. coli* OxyR [16,33]. The hydrophobic side chain of Met206 is inserted into the hydrophobic cavity formed by residues Leu174, Tyr200, Tyr205, Leu209, Ile210, Pro248, Tyr250, and Ile273; this cavity is predicted to be disturbed upon the oxidation of Met206 into methionine sulfoxide, causing the overall structural rearrangement of the HypT homodimer, as shown by the Met206-SOH-mimicking M206Q structure (Figure 4) [33]. The crystal structure of the RD domain in HypT M206Q reveals that a concerted movement of secondary structural elements occurs, and that the small hydrophobic cavity created by the backbone amides of Met206 collapses as the side chain of Met206 moves away from the hydrophobic cavity. The structural change would eventually lead to an overall conformational rearrangement of the tetrameric assembly, and the regulation of the DNA-binding properties of the protein, leading to the upregulation of the target genes, as shown by qRT-PCR analysis [33], but further structural analyses are required to elucidate the atomic details.

## 4. Iron-Based Redox Sensors

### 4.1. Sensing by Fe–S Cluster: SoxR

*E. coli* SoxR (superoxide response), which is a member of the MerR family transcription activators of the *soxRS* regulon, senses oxidative stress through the oxidation state of its iron–sulfur cluster [40]. SoxR regulates the expression of *soxRS* regulon genes by binding to the *soxS* promoter [40,41,42]. Oxidative stress induces the oxidation of iron–sulfur metal centers, leading to SoxR-induced upregulation of *soxS* expression, subsequently increasing the levels of SoxS protein to activate the transcription of various *soxRS* regulon genes [43,44,45]. Interestingly, both oxidized and reduced SoxR bind to *soxS* promoter DNA with a similar affinity, according to the electrophoretic mobility shift assays [46]. However, only oxidized SoxR is able to activate the transcription of *soxS*, suggesting that the structural transition from a reduced SoxR–*soxS*-promoter DNA complex to an oxidized SoxR–*soxS*-promoter DNA complex may mediate the activation of RNA polymerase [43,46].

**Figure 5:**
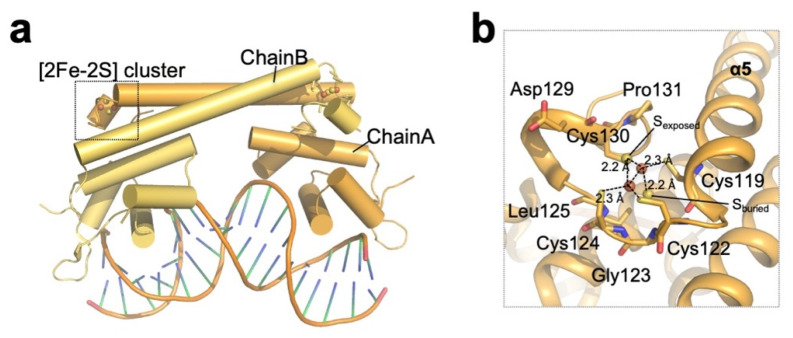
**Structure of redox-sensing [2Fe–2S] cluster-containing SoxR.** (**a**) Overall structure of the oxidized (activated) *E. coli* SoxR homodimer (PDB ID: 2ZHG; chain A; orange, chain B; yellow) bound to DNA. (**b**) Close-up view of the [2Fe–2S] cluster of *E. coli* SoxR chain A. Cysteine residues coordinating the cluster are indicated, and the residues involved in the asymmetric environment are shown as sticks.

The *E. coli* SoxR protein is dimeric, and each subunit consists of an N-terminal DBD, a dimerization helix (α5), and a C-terminal domain with a [2Fe–2S] cluster-binding domain, which is essential for SoxR activity [47] (Figure 1, Table 1). The dimerization helix forms an antiparallel coiled coil, and the [2Fe–2S] cluster-binding domain, which resides in the C-terminus of the helix, is stabilized through interactions with the DNA-binding domain of the other subunit (Figure 5a). The Cys119–Pro131 sequence in each subunit comprises the [2Fe–2S] cluster-binding domain (a CX2CXCX5C motif), and the cluster is directly coordinated by four conserved cysteines (Cys119, Cys122, Cys124, and Cys130) [48] (Figure 5b). The structure of the coordinating [2Fe–2S] cluster in the SoxR protein is very different from the typical [Fe–S] structure found in other [Fe–S] cluster-coordinated proteins [49]. The typical [Fe–S] clusters and their ligand atoms in proteins, such as ferredoxins, are often buried within the protein and shielded from solvent [50,51]. In contrast, the [2Fe–2S] cluster in SoxR is partially exposed to solvent and is asymmetrically bound by protein ligands [47]. Buried sulfur ions (Sburied) make extensive van der Waals contact and establish several hydrophobic interactions, while exposed sulfur ions (Sexposed) establish only a few interactions with the protein. This asymmetric environment plays a key role in inducing structural changes in SoxR depending on the redox state of the [2Fe–2S] cluster. The reduction of oxidized clusters ([2Fe–2S]2+ with two ferric ions) to reduced clusters ([2Fe–2S]+ with one ferric ion and one ferrous ion) by one electron confers an additional negative charge on the sulfur ion, attracting the backbone amides clustered around Sburied. This clustering increases the electrostatic repulsion between the backbone amides and positively charged C-terminal region of SoxR to create an “open” conformation. As the C-terminal region of SoxR interacts directly with the N-terminal DNA-binding domain of the other subunit, conformational changes in the cluster-binding region are thought to drive distortion in the DNA, inhibiting RNA polymerase from initiating transcription.

Interestingly, SoxR has been shown to sense nitric oxide through distinct activation mechanisms that differ from the oxidation/reduction of [2Fe–2S] clusters [52]. Nitric oxide directly modifies the [2Fe–2S] clusters to generate dinitrosyl–iron–dithiol clusters, and this modification activates the transcriptional activity of the SoxR protein. A more detailed structural and functional description of the direct nitrosylation of [2Fe–2S]-mediated signal transduction awaits further study.

### 4.2. Sensing by Fe-Catalyzed Histidine Oxidation: PerR

*B. subtilis* exhibits an adaptive response to peroxide that is controlled mainly by PerR, σ^B^, and OhrR transcription factors [53]. PerR is a major metal-dependent peroxide-sensing transcriptional regulator in *B. subtilis* and in several pathogenic bacteria [54,55]. Derepression of the PerR regulon (which includes genes encoding catalase (KatA), alkyl hydroperoxide reductase (AhpCF), heme biosynthesis enzymes (HemAXCDBL), a Zn^2+^ uptake P-type ATPase (ZosA), and the Dps-like DNA-binding protein MrgA) leads to the detoxification of peroxides and the minimization of the damage caused by intracellular Fe-catalyzed redox reactions. PerR is a member of the Fur family of transcriptional regulators, which sense peroxides by Fe-catalyzed histidine oxidation [56]. PerR is a dimeric protein, with each subunit composed of an N-terminal winged helix-turn-helix DNA-binding domain and a C-terminal domain involved in dimerization (Figure 1). The C-terminal dimerization domain contains two metal-binding sites: a Zn^2+^-binding site that has a structural role and an Fe^2+^- or Mn^2+^-binding site that has a regulatory role [57].

Several structural studies have elucidated how PerR responds to peroxide stresses and undergoes structural changes to induce peroxide stress responses [58,59,60,61,62,63,64] (Table 1). In *B. subtilis* PerR, four cysteine residues (Cys96, Cys99, Cys136 and Cys139) cluster to form a Zn2+-binding site, while three histidine and two aspartate residues (His3Asp2; His37, Asp85, His91, His93 and Asp104) coordinate the regulatory metal ion Fe2+ or Mn2+ [62,63]. Zn^2+^ ion binding to the structural site is required for the stable dimerization of the PerR protein. The four cysteine residues forming the structural site are exceptionally resistant to H2O2 and are not involved in peroxide sensing, presumably because the residues are protected by the coordinated Zn2+ ion [65].

**Figure 6:**
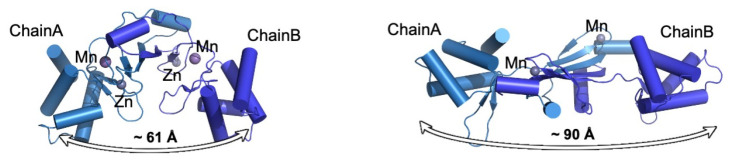
**Reorganization of the overall dimeric assembly of *B. subtilis* PerR upon oxidation.** PerR-Zn-Mn (left, PDB ID: 3F8N) adopts a curved conformation, and PerR-Zn-apo (right, PDB ID: 2FE3) adopts a relatively flattened structure. The oxidation of His37 to 2-oxo-histidine leads to the dissociation of His37 from the regulatory metal, preventing PerR from adopting a DNA-binding compatible curved shape. The distance denotes the distance between the two DBDs in the PerR homodimer (measured as the distance between Asn53/Asn53′ Cα atoms).

The binding of the metal to the regulatory site and the incorporation of one oxygen into either His37 or His91 are required for DNA derepression. His3Asp2 coordinates Fe2+ or Mn2+ ions into a pentacoordinate square pyramidal geometry, with His93 at one apical position and a H2O2 molecule at the opposite apical position of the pyramid. His37, Asp85, His91, and Asp104 are arranged in an equatorial plane [62,63]. The regulatory site is surrounded by hydrophilic molecules, explaining its preference for H_2_O_2_ over organic hydroperoxides. Comparisons of the structures of *B. subtilis* PerR-Zn-apo (PDB ID: 2FE3) and PerR-Zn-Mn (PDB ID: 3F8N) reveal remarkable differences, mainly in the relative positions of the DNA-binding domains. PerR-Zn-apo molecules have a relatively flattened conformation compared to that of the PerR-Zn-Mn structure; thus, the DNA-binding domains are farther apart in the apo structure, making it seemingly incompatible with DNA binding. The binding of His37, which resides in the N-terminal DNA-binding domain, to the regulatory metal induces the reorientation of the N-terminal-binding domain, and subsequently, the overall PerR protein dimer adopts a banana-like curved structure, which is compatible with DNA binding (Figure 6) [61]. The oxidation of His37 to 2-oxo-histidine leads to the dissociation of the protein from the regulatory metal, preventing PerR from adopting a DNA-binding-compatible curved shape. The oxidation of His91 also leads to the dissociation of the protein from DNA through a different mechanism. Electron paramagnetic resonance (EPR) analysis showed that the oxidation of His91 simply abolishes the metal binding in the regulatory site, and PerR cannot adopt a banana-shaped curved conformation compatible with DNA binding [61]. In summary, PerR utilizes two histidine residues to sense oxidative stresses to cope with peroxide stress, each with a distinct structural response mechanism.

### 4.3. Sensing by Heme-Based Redox Sensor: DosS/DosT-DosR System

Importantly, bacterial heme-based sensor proteins exploit the redox chemistry of the heme to sense environmental gases and the intracellular redox state of the bacterial cell. Heme (iron–protoporphyrin IX complex) is a ubiquitous molecule that is essential for the critical functions of aerobic cells [66]. Hemoglobin and myoglobin transport and store oxygen, respectively, whereas cytochromes are involved in electron transport, energy generation, and chemical transformation. Heme-based sensors of redox stress are widespread in nature. Heme is most often bound to a PAS domain or GAF domains.

In *Mycobacterium tuberculosis*, the Dos two-component regulatory system is critical for sensing hypoxia, nitric oxide (NO), and carbon monoxide (CO), which are typically associated with the onset of latent tuberculosis [67,68]. *M. tuberculosis* can adapt to the hypoxic environment, NO, and CO via a three-component regulatory system: the DosS/DosT and DosR system. This system consists of two sensor histidine kinases, DosS and DosT, and the dormancy survival transcriptional regulator DosR, which controls the expression of a set of at least 48 genes involved in the redox response [69,70,71,72], which allows *M. tuberculosis* to adapt to anaerobic dormant conditions and tolerate antibacterial agents [73].

DosS and DosT histidine kinases both consist of an N-terminal tandem GAF domain (cGMP, adenyl cyclase, and FhlA; GAF-A and GAF-B), followed by a sensor kinase domain and a C-terminal ATPase domain. The GAF-A domain binds a heme through a conserved histidine residue (His149 in DosS; His147 in DosT), while the adjacent GAF domain (GAF-B) has been shown to not bind heme or cyclic nucleotides, and its function has yet to be discovered (Figure 1) [74,75,76]. The oxidation and ligation states of heme iron modulate the autokinase activity of DosS and DosT sensor kinases. Under hypoxic conditions or upon binding NO or CO, the deoxy ferrous form of DosS/DosT is autophosphorylated at a conserved histidine (His395/His397 in DosS; His392/His394 in DosT) in the kinase domain [69]. Once phosphorylated, DosS/T subsequently phosphorylates an aspartate residue (Asp54) in the regulatory domain of DosR, the response regulator [69]. This modification results in the binding of DosR to DNA upstream of hypoxic response genes and thus activates the dosR regulon. On the other hand, upon binding oxygen, DosS/DosT is not autophosphorylated, and thus represses the expression of the DosR regulon [71].

**Figure 7:**
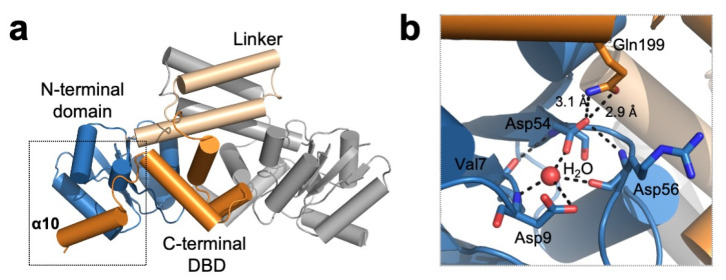
***M tuberculosis* DosR is in an autoinhibited state.** (**a**) Overall structure of *M. tuberculosis* DosR (chain A: blue, ivory, orange; chain B: gray. PDB ID: 3C3W). The N-terminal domain (blue) interacts with the C-terminal DNA-binding domain (orange), suggesting that DosR adopts an autoinhibited state. (**b**) The DosS/T kinase substrate Asp54 plays a critical role in stabilizing autoinhibitory interactions by forming a hydrogen bond with Gln199 in the α10 helix. Residues involved in hydrogen bonding with Asp54 are shown as sticks.

Several crystallographic studies showed the detailed mechanism of how the phosphorylation of catalytic Asp54 in DosR leads to the structural rearrangement that induces DosR–DNA complex formation (Table 1). DosR comprises three domains: an N-terminal regulatory domain (residues 1–97) where DosS/T kinase substrate residue (Asp54) resides; a middle linker domain (residues 98–149); and a C-terminal helix-turn-helix DNA-binding domain (residues 150–210) (Figure 7) [77]. Interestingly, the C-terminal DNA-binding domain folds back to interact with an N-terminal regulatory domain, suggesting that unphosphorylated DosR is in an autoinhibitory state incapable of binding DNA [77,78]. The autoinhibitory interaction is stabilized though extensive hydrogen bonding and hydrophobic interactions between two domains with a surface area of ~2500 A2. Importantly, Asp54 plays a critical role in maintaining the structural rigidity of the autoinhibitory state by forming a hydrogen bond with Asn199 in the α10 helix of the C-terminal DNA-binding domain (Figure 7). The phosphorylation of Asp54 is assumed to stimulate the release of autoinhibitory interactions and results in an active/open conformation enabling DosR to interact with cognate DNA [77,78].

## 5. Nucleotide-Sensing Redox Sensors

### 5.1. Sensing NAD+/NADH: Rex

As electron carriers, nicotinamide adenine dinucleotides (NAD and NADH) play essential roles, directly and indirectly, in numerous biological processes in both eukaryotes and bacteria [79,80,81]. During bacterial aerobic respiration, NAD+ is reduced to NADH through glycolysis and the tricarboxylic acid (TCA) cycle [82]. NADH is then oxidized back to NAD+ by the mitochondrial electron transport chain complex, with oxygen functioning as the terminal electron acceptor mediating electron flow [83]. The generated electrochemical gradient across the mitochondrial membrane powers ATP synthase to produce energy [84]. NADH levels increase when the cells are deprived of oxygen or the electron transport chain shows impaired activity, requiring bacteria to continuously monitor and carefully maintain the NADH:NAD+ balance using transcriptional regulators [85,86,87].

A redox-sensing repressor (Rex) was first identified in the Gram-positive antibiotic-producing bacterium *Streptomyces coelicolor* as a redox-sensing transcriptional regulator [88], and later was identified in numerous other model microorganisms, including *Staphylococcus aureus, Bacillus subtilis, Enterococcus faecalis,* and *Streptococcus mutans* ([89,90,91,92]). Rex regulates the transcription of respiratory genes, such as NADH dehydrogenase (*nuoA-nuoN*)*,* heme biosynthetic enzymes (*hemACD*)*,* cytochrome oxidase (*cydABCD*), and Rex itself, in response to changes in the intracellular NADH:NAD+ balance [88]. Under aerobic conditions, Rex binds to its cognate DNA, repressing transcription. Various biochemical studies including SPR analysis have shown that the elevated NADH levels lead to the prevention of Rex binding DNA, derepressing the transcription of redox-responsive genes [88,93,94,95].

**Figure 8:**
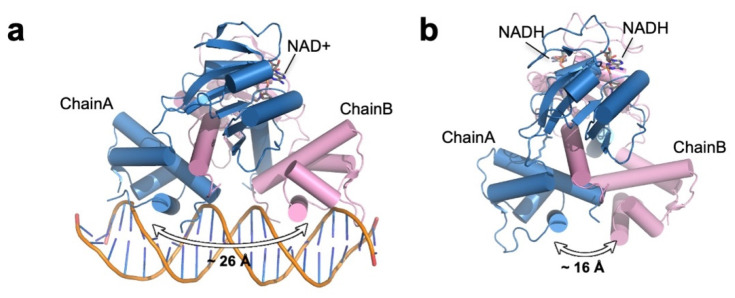
Structural comparison between Rex in complex with (**a**) NAD+ (left, the *T. thermophilus* Rex–NAD+–DNA complex; PDB ID: 3IKT) and (**b**) NADH (right, the *T. aquaticus* Rex–NADH complex; PDB ID: 1XCB). The distance denotes the distance between the two HTH motifs in the Rex homodimer (measured as the distance between Lys47/Lys47′ Cα atoms).

Various structural studies of Rex in complex with NADH, NAD^+^, its cognate DNA or in the apo state revealed how Rex translates the NADH:NAD+ ratio into a structural conformational change to control the binding affinity between the protein and DNA [95,96,97,98,99] (Table 1). The structural organization and overall structural characteristics of Rex in various organisms are very similar to each other. Rex is a homodimer and can be divided into two functional domains: an N-terminal DNA-binding domain with a winged helix-turn-helix and a C-terminal NAD+/NADH-binding domain that adopts a characteristic Rossman fold (consecutive alternating β-strands and α-helices that form a layer of β-sheets with one (or two) layer(s) of α-helices) (Figure 1). Regardless of its ligand-binding state, Rex is a homodimer, and the dimeric structure is stabilized by a domain-swapped helix. The last helix of the C-terminal domain inserts deep into the hydrophobic cavities formed by the residues of the N-terminal DNA-binding domain and C-terminal domain of the opposing subunit. These extensive hydrophobic interactions stabilize the homodimeric structure of Rex. In the structures of Rex in complex with NAD+ or NADH, the NAD+/NADH-binding pocket can be divided into two parts: (i) a P-loop containing a hydrophobic pocket where the ADP moiety binds, and (ii) a pocket near the dimeric interface formed by the N-terminus of the domain-swapped α-helix and the α5/β4 loop of the opposing subunit, where nicotinamide and the N-ribose moiety bind [95,96,97,98]. In both NAD+- and NADH-bound structures, the ADP moiety is fixed stably in the pocket and remains essentially the same, whereas the nicotinamide and N-ribose moieties show different binding patterns (Figure 8). In the structure of the NAD+–Rex complex, the carboxamide nitrogen atom and N-ribose group for hydrogen bonds with the highly conserved Ala and Tyr residues of the other subunit, respectively (Ala 94 in *T. aquaticus* Rex and Ala96 in *T. maritima* Rex; Tyr98 in *T. aquaticus* Rex and Tyr100 in *T. maritima* Rex) [95,98]. In comparison, the reduced nicotinamide of NADH is flipped when the carboxamide group forms hydrogen bonds with the main chain atoms of the N-terminal residues in the domain-swapped α-helix (Phe189 in *T. aquaticus* Ile190, 192 in *T. maritima* Rex) [96,98]. As the domain-swapped helix interacts with the N-terminal DNA-binding domain of the other subunit, the structural rearrangement in the NAD+/NADH-binding pocket affects the DNA binding domain, ultimately altering the conformation regulating the DNA-binding properties (Figure 8).

### 5.2. Sensing FAD+/FADH: NifL-NifA System

Flavin mononucleotide (FMN) and flavin adenine dinucleotide (FAD) are two cofactors that ensure the functionality of enzymes involved in fundamental biochemical processes, including oxidative metabolism of carbohydrates, amino acids, fatty acids; mitochondrial electron transport; and redox homeostasis, by serving as either electron acceptors in the oxidized form or as electron donors in the reduced form [100,101]. To date, several flavin-based sensor proteins that sense FAD/FMN have been reported [102,103,104]. Among these proteins, the NifL–NifA regulatory system is one of the best-characterized transcriptional regulation systems. Nitrogen-fixing prokaryotes (diazotrophs) are capable of fixing atmospheric nitrogen to ammonia with nitrogenase to survive and proliferate under nitrogen deprivation conditions. Nitrogen fixation based on nitrogenase requires large amounts of ATP and leads to oxidative stresses that fix atmospheric nitrogen [105,106]. Because nitrogenase can also be readily oxidized by oxygen, which leads to irreversible inactivation and blocks nitrogen fixation, diazotrophic bacteria must delicately sense oxidative stresses and employ protective strategies to ensure that the nitrogenase enzyme remains active. Within the γ subgroup of proteobacteria, including *Azotobacter vinelandii*, the regulatory protein NifL senses and consolidates the redox, carbon, and fixed nitrogen states and controls the activity of the transcriptional activator NifA [106]. The genes encoding NifA and NifL are organized in an operon, and NifL binds to NifA and inhibits its transcription-activating function by forming a stable protein complex when environmental circumstances are not suitable for nitrogen fixation. The binding affinity between NifA and NifL is controlled by NifL in response to the redox status of the cell [107,108].

*A. vinelandii* NifL is a tetrameric protein [109] in which each monomer can be divided into three discrete functional domains: the N-terminal-sensing domain, a C-terminal kinase-like domain and glutamine-rich linker domain connecting the N-terminal and C-terminal domains (Figure 1). The N-terminal domain contains tandem PAS domains, and the first N-terminal PAS domain (PAS1) binds an FAD that is responsive to oxidative status, and thus is necessary for sensing redox stress [110], whereas the PAS2 domain is proposed to transduce the redox signal perceived by the PAS1 domain by changing the quaternary structure of the protein [111]. The C-terminal domain contains a conserved H motif (H domain) and a nucleotide-binding GHKL (Gyrase, Hsp90, Histidine kinase, MutL) domain that are structurally related to the ATPase domains of histidine kinase. Despite the structural similarities, NifL does not exhibit kinase activity, regulating its partner NifA by direct protein–protein interactions, not by phosphorylation [112].

**Figure 9:**
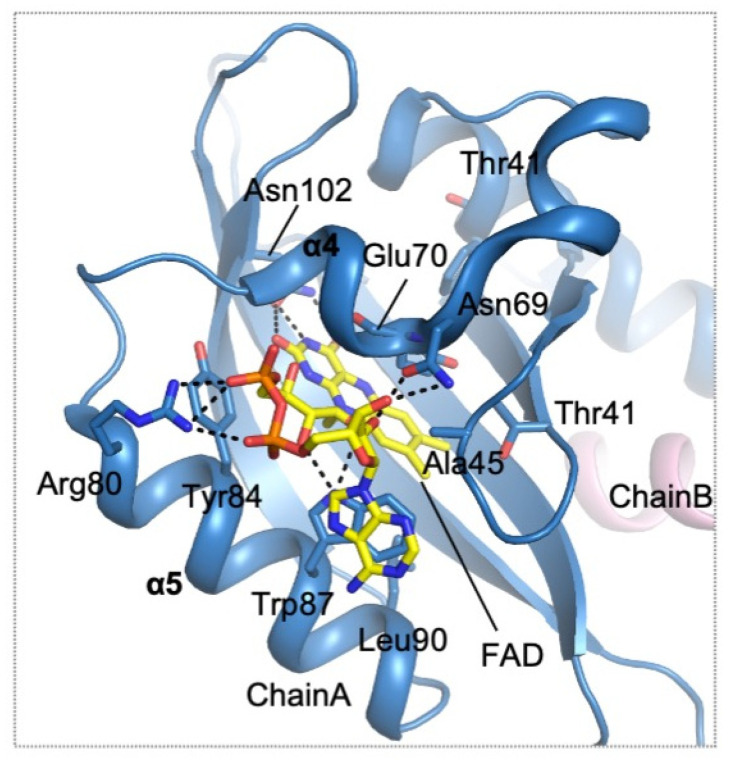
**Residues involved in FAD sensing by *A. vinelandii* NifL.** Ribbon diagram showing the structure of NifL (chains A and B are colored blue and pink, respectively) in complex with FAD (carbon atoms are colored yellow). The side chains interacting with NifL are shown as sticks and are colored by atom type. Dotted lines denote the hydrogen bonds established between NifL and bound FAD.

Although the atomic resolution of the full-length protein has not been elucidated, the crystal structure of the *A. vinelandii* NifL PAS1 domain complexed with FAD cofactor has offered insight into FAD cofactor-based redox sensing [113] (Table 1, Figure 9). The PAS1 domain of NifL displays a canonical PAS domain fold consisting of five-stranded β sheets flanked by three α helices. The PAS1 domain is a dimeric protein in which a single oxidized FAD is bound within the core of each monomer through extensive hydrogen bonding and several hydrophobic interactions. The isoalloxazine ring portion of FAD is stabilized through hydrogen bonds formed with the side chain of Asn102 and the hydroxyl group of Tyr83. The residues involved in the hydrophobic interactions with the isoalloxazine ring include Thr41, Ala45, Glu70, Trp87 and Leu90 [113] (Figure 9). The ribose and adenine moiety of FAD is mainly stabilized through interactions with Trp87 and Arg80. The side chain indole ring of Trp87 establishes a pi-stacking interaction with the base moiety of the adenine moiety of the bound FAD molecule [113]. The positively charged side chain of Arg80 forms a salt bridge with O1P phosphate. Although the FAD molecule is deeply buried inside the core of the PAS domain, two cavities are present near the N5 and O4 atoms of the bound molecule. These cavities might provide room and passage for the formation of hydroperoxyl intermediate species and oxidative stress-inducing molecules such as hydrogen peroxide. Oxidation/reduction of the N5 atom of the isoalloxazine ring in FAD would induce the reorganization of the hydrogen bonding network and hydrophobic interactions, subsequently changing the overall conformation of the protein. The redox signal perceived by the first PAS domain must be somehow transmitted to the C-terminal domain of NifL, the domain critical for the interaction with NifA, to elicit a response. Further structural study on other portions of the NIfL, as well as the NifA regulatory protein, will provide molecular details on how bacteria capture FAD/FADH and respond to oxidative stress.

## 6. Concluding Remarks

Redox sensing and adaptation to oxidative stress are critical for the life of bacteria. Bacteria respond to oxidative stress with a wide range of adaptive changes, involving changes in gene expression and metabolism to redirect sources into processes that are essential for survival, not growth. Therefore, redox sensors are especially important in the virulence and dormancy of pathogenic bacteria, as they typically confer resistance to antibiotics [73,114,115,116]. Although most of the bacterial redox sensors discussed herein contain a sensory domain (that recognizes the redox signal directly) and an effector domain (that exerts the response, generally by changing its affinity toward DNA) in a single polypeptide chain, several bacterial redox sensors consist of two components, suggesting that redox control can be mediated in a sophisticated way. As structural information is lacking for two-component sensors, one of the future challenges is to gain an understanding of their molecular details.

## Figures and Tables

**Table 1:** **Bacterial redox sensors**. Abbreviations: RD, Regulatory domain; DBD, DNA binding domain; FL, Full-length; *P. gingivalis*, *Porphyromonas gingivalis*; *P.aeruginosa*, *Pseudomonas aeruginosa*; *E. coli*, *Escherichia coli*; *C glutamicum*, *Corynebacterium glutamicum*; *N. meningitidis*, *Neisseria meningitidis*; *V. vulnificus*, *Vibrio vulnificus*; *B. subtilis*, *Bacillus subtilis*; *S. typhimurium*, *Salmonella typhimurium*; *L. interorgans*, *Leptospira interrogans*; *S. pyogenes*, *Streptococcus pyogenes*; *C. jejuni*, *Campylobacter jejuni*; *M. tuberculosis*, *Mycobacterium tuberculosis*; *A.vinelandii*, *Azotobacter vinelandii*; *T. aquaticus*, *Thermus aquaticus*; *T. thermophilus*, *Thermus thermophilus*; *T. maritima*, *Thermotoga maritima*.

Sensing Mechanism	Sensor	Organism	PDB Code	Structural Feature	Reference
***Thiol based redox sensors***					
**Reversible disulfide-bond Formation**	OxyR	*E. coli*	1I69	C199S (RD)	[16]
			1I6A	Oxidized (RD)	
		*C. glutamicum*	6G1D	C206S (FL)	[17]
			6G4R	C206S-H_2_O_2_ (FL)	
			6G1B	Oxidized (FL)	
		*P. aeruginosa*	4Y0M	Reduced (RD)	[19]
			4XWS	C199D (RD)	
			4X6G	C199D-H_2_O_2_ (FL)	
		*P. gingivalis*	3HO7	Oxidized (RD)	[36]
			3UKI	Reduced (RD)	
			3T22	C199S (RD)	
		*N. meningitidis*	3JV9	Reduced	[37]
		*V. vulnificus (OxyR2)*	5X0Q	Cl-bound	[38]
			5B70	E204G mutant	
			5B7D	Sulfate-bound	
**Disulfide bond/Thiol alkylation**	YodB	*B. subtilis*	5HS9	Oxidized (quinone bound)	[28]
			5HS8	Oxidized (Disulfide bonded)	
			5HS7	Reduced	
***Methionine based redox sensors***					
**Methionine oxidation**	HypT	*S. typhimurium*	5YDV	HOCl bound (RD)	[33]
			5YDW	FL	
			5YDO	Apo form (RD)	
			5YEZ	M206Q mutant (RD)	
			5YER	Bromide bound (RD)	
***Iron dependent redox regulator***					
**Fe-S cluster**	SoxR	*E. coli*	2ZHH	Apo form	[47]
			2ZHG	DNA complex	
**Fe-mediated His oxidation**	PerR	*L. interrogans*	5NL9	PerR-Zn-apo	[58]
		*S. pyogenes*	4LMY	PerR-Zn-Zn	[59]
			4I7H	PerR-Zn-apo	[60]
		*B. subtilis*	2RGV	PerR-Zn-apo(2-oxo-His)	[61]
			3F8N	PerR-Zn-Mn	[62]
			2FE3	PerR-Zn-Mn	[63]
		*C. jejuni*	6DK4	PerR-Zn-Mn	[64]
**Haem**	DosR	*M. tuberculosis*	3C57	DBD	[77]
			3C3W	FL	
			1ZLJ	DBD	[78]
			1ZLK	DNA complex	
***Sensing nucleotide***					
**Sensing NAD/NADH ratio**	Rex	*T. thermophilus HB27*	3IL2	DNA complex	[95]
			3IKT	DNA-NAD+ complex	
			3IKV	R90D mutant	
		*T. aquaticus*	1XCB	NADH complex	[96]
		*B. subtilis*	2VT2	Apo form	[97]
			2VT3	ATP complex	
		*T. maritima MSB8*	5ZZ5	Apo form	[98]
			5ZZ6	NAD+ complex	
			5ZZ7	NADH complex	
		*T. thermophilus HB8*	2DT5	NAD+ complex	[99]
**Sensing FAD/FADH ratio**	NifL	*A. vinelandii*	2GJ3	PAS domain	[113]
